# Supplementary material for: Biochemical and structural characterization of an inositol pyrophosphate kinase from a giant virus
Source: EMBO J. 2024 Jan 12;43(3):7. doi: 10.1038/s44318-023-00005-0 (PMC10897400; doi:10.1038/s44318-023-00005-0)
Supplement: Supplementary file 1 — Table EV1 [file 44318_2023_5_MOESM1_ESM.docx]

**Table EV1** Data collection and refinement statistics

| PDB Accession IDs | 8T8U | 8T8V | 8T8W | 8T8X | 8T8Y | 8T8Z |
| --- | --- | --- | --- | --- | --- | --- |
| Protein | *Tv*IPK (1-265) | *Tv*IPK (17-265) | *Tv*IPK (17-265) | *Tv*IPK (17-265) | TvIPK (17-265) | *Tv*IPK (17-265) |
| Ligand(s) | ADP  Pi | ADP  Pi | ATP  Pi | AMP-PNP  Pi | ADP  *myo*-(1,4,5)IP_3_ | ADP  *myo*-(1OH)IP_5_ |
| Metal Ion | Mg | Mg | Cd | Cd | Mg | Mg |
| Space group | C222_1_ | C222_1_ | C222_1_ | C222_1_ | C222_1_ | C222_1_ |
| Cell parameters *a*, *b*, *c* (Å) | 61.9, 104.9, 102.4 | 61.7, 104.7, 102.5 | 63.1, 104.2, 102.4 | 63.5, 103.4, 102.7 | 64.6, 100.6, 105.0 | 63.8, 103.8, 102.7 |
| Resolution (Å) | 50.0-2.0 | 50.0-1.95 | 50.0-2.0 | 50.0-2.6 | 50.0-2.50 | 50.0-2.60 |
| Rmeas* | 0.086 (0.923) | 0.057 (0.852) | 0.076 (0.837) | 0.11 (0.984) | 0.152(0.894) | 0.098(0.748) |
| *I/σ I** | 24.9 (1.94) | 24.3 (1.97) | 34.2 (3.2) | 19.9 (2.2) | 16.0 (3.2) | 16.5 (1.97) |
| Completeness (%)* | 99.6 (97.8) | 99.6 (98.5) | 99.9 (99.9) | 99.5 (100) | 99.0 (100) | 99.4 (98.8) |
| Redundancy* | 8.5 (8.5) | 3.8 (3.8) | 12.9 (11.9) | 7.1 (6.9) | 8.0 (8.3) | 7.1 (6.9) |
| Refinement |  |  |  |  |  |  |
| Resolution (Å)* | 2.01 (2.06) | 1.96 (2.01) | 2.0 (2.05) | 2.60 (2.66) | 2.50 (2.57) | 2.60 (2.67) |
| No. reflections | 19768 | 20981 | 21798 | 9632 | 11245 | 9615 |
| *R*_work_* | 17.3 (22.8) | 19.9 (26.4) | 15.9 (23.9) | 17.8 (25.5) | 17.3 (22.7) | 17.4 (27.4) |
| *R*_free_* | 20.4 (26.2) | 23.2 (33.9) | 19.5 (25.2) | 22.9 (22.4) | 22.3 (36.5) | 23.4 (22.4) |
| *No*. *atoms* |  |  |  |  |  |  |
| Protein | 1779 | 1784 | 1782 | 1772 | 1764 | 1772 |
| Ligand/ion | 34 | 39 | 75 | 46 | 53 | 60 |
| Solvent | 168 | 159 | 185 | 73 | 117 | 58 |
| B-factors (Å^2^) |  |  |  |  |  |  |
| Protein | 28.6 | 21.3 | 28.9 | 41.1 | 39.0 | 41.0 |
| Ligand/ion | 26.7 | 23.0 | 31.0 | 64.4 | 30.4 | 59.5 |
| Solvent | 40.6 | 32.3 | 39.2 | 37.4 | 36.7 | 37.1 |
| R.M.S. deviations |  |  |  |  |  |  |
| Bond length (Å) | 0.004 | 0.004 | 0.013 | 0.008 | 0.005 | 0.009 |
| Bond angle (°) | 1.24 | 1.25 | 1.74 | 1.62 | 1.44 | 1.62 |

**Table EV1** Continue

| PDB Accession IDs | 8T90 | 8T91 | 8T92 | 8T93 | 8T95 | 8T96 | 8T97 |
| --- | --- | --- | --- | --- | --- | --- | --- |
| Protein | TvIPK (17-265) | *Tv*IPK (17-265) | TvIPK (17-265) | TvIPK (17-265) | TvIPK (17-265) | TvIPK (17-265) | TvIPK (17-265) |
| Ligand(s) | ADP  *myo*-(3OH)IP_5_ | ADP  *myo*-IP_6_ | ADP  D-*scyllo*- (1,2,3,4)IP_4_  Pi | ADP  L-*scyllo*- (1,2,3,4)IP_4_ | ADP  *scyllo*-(1,2,4,5)IP_4_ | ADP  *scyllo*-IP_5_  Pi | ADP  *scyllo*-IP_6_  Pi |
| Metal Ion | Mg | Mg | Mg | Mg | Mg | Mg | Mg |
| Space group | C222_1_ | C222_1_ | C222_1_ | C2221 | C2221 | C2221 | C2221 |
| Cell parameters  a, b, c (Å), | 64.3, 103.1, 103.9 | 64.5, 103.6, 103.4 | 62.5, 104.1, 103.8 | 64.7, 103.4, 103.7 | 64.3, 103.1, 104.1 | 63.6, 103.4, 104.2 | 63.2, 103.1, 103.8 |
| Resolution (Å) | 50.0-2.30 | 50.0-2.55 | 50.0-2.30 | 50.0-1.95 | 50.0-2.60 | 50.0-2.35 | 50.0-2.45 |
| Rmeas* | 0.078(0.759) | 0.106(0.589) | 0.143 (0.933) | 0.065(0.785) | 0.095(0.891) | 0.193(0.691) | 0.088(0.762) |
| *I/σ I** | 30.4 (3.5) | 19.0 (2.6) | 19.5 (2.7) | 38.5 (3.6) | 30.7 (3.7) | 19.6 (2.1) | 26.7 (2.3) |
| Completeness (%)* | 99.9 (99.9) | 98.8 (96.7) | 99.2 (99.2) | 99.3 (99.5) | 99.9 (100) | 92.8 (77.1) | 99.5 (98.7) |
| Redundancy* | 12.2 (11.7) | 8.1 (6.1) | 19.8 (15.6) | 11.1 (10.8) | 13.2 (13.8) | 17.5 (9.1) | 9.1 (8.4) |
| Refinement |  |  |  |  |  |  |  |
| Resolution (Å)* | 2.30 (2.36) | 2.56 (2.63) | 2.30 (2.36) | 1.95 (2.0) | 2.61 (2.68) | 2.36 (2.42) | 2.46 (2.52) |
| No. reflections | 14593 | 10547 | 13443 | 24214 | 10209 | 12750 | 11078 |
| *R*_work_ (%)* | 17.1 (22.3) | 18.9 (33.6) | 16.8 (20.4) | 16.4 (22.8) | 16.8 (24.8) | 16.8 (26.9) | 18.0 (24.4) |
| *R*_free_ (%)* | 21.7 (22.6) | 23.7 (35.9) | 22.8 (28.8) | 19.6 (25.8) | 22.9 (26.3) | 22.6 (21.2) | 22.8 (29.1) |
| *No*. *atoms* |  |  |  |  |  |  |  |
| Protein | 1764 | 1772 | 1764 | 1776 | 1764 | 1770 | 1766 |
| Ligand/ion | 60 | 64 | 62 | 56 | 56 | 66 | 69 |
| Solvent | 105 | 87 | 98 | 222 | 79 | 115 | 67 |
| B-factors (Å^2^) |  |  |  |  |  |  |  |
| Protein | 33.9 | 37.7 | 32.1 | 23.1 | 42.1 | 51.0 | 38.9 |
| Ligand/ion | 54.0 | 55.9 | 39.4 | 29.3 | 82.4 | 75.9 | 75.2 |
| Solvent | 38.4 | 36.0 | 35.7 | 35.7 | 42.0 | 53.5 | 39.7 |
| R.M.S. deviations |  |  |  |  |  |  |  |
| Bond length (Å) | 0.011 | 0.006 | 0.012 | 0.015 | 0.009 | 0.007 | 0.010 |
| Bond angle (°) | 1.75 | 1.50 | 1.85 | 1.87 | 1.67 | 1.67 | 1.84 |

**Table EV1** Continue

| PDB Accession IDs | 8TF9 | 8TFA | 8T98 | 8T99 |
| --- | --- | --- | --- | --- |
| Protein | DIPP1 (1-148) | DIPP1 (1-148) | DIPP1 (1-148) | DIPP1 (1-148) |
| Ligand | *myo*-5-PP-IP_5_ | *myo*-5-PP-(1,3,4,6)-IP_4_ | *scyllo*-3-PP- (1,2,4,5)IP_4_ | L-*scyllo*-1,4-[PP]_2_-(2,3)IP_2_ |
| Space group | P2_1_2_1_2_1_ | P2_1_2_1_2_1_ | P2_1_2_1_2_1_ | P2_1_2_1_2_1_ |
| Cell parameters *a*, *b*, *c* (Å), | 45.0, 59.6, 62.7 | 45.3, 59.6, 62.7 | 46.1, 59.6, 62.4 | 45.8, 59.6, 62.7 |
| Resolution (Å) | 50.0-1.55 | 50.0-1.4 | 50.0-1.3 | 50.0-1.5 |
| Rmeas* | 0.144(0.464) | 0.092(0.681) | 0.071 (0.466) | 0.091(0.518) |
| *I/σ I** | 24.9 (2.1) | 39.0 (2.1) | 36.6 (2.5) | 25.4 (2.7) |
| Completeness (%)* | 96.8 (82.9) | 97.2 (79.9) | 98.0 (82.8) | 94.2 (77.2) |
| Redundancy* | 9.4 (4.9) | 10.9 (7.8) | 10.2 (9.0) | 10.0 (8.3) |
| Refinement |  |  |  |  |
| Resolution (Å)* | 1.55 (1.59) | 1.40 (1.44) | 1.30 (1.33) | 1.50 (1.54) |
| No. reflections | 23137 | 31613 | 40052 | 25145 |
| *R*_work_ (%)* | 16.1 (29.7) | 15.4 (25.4) | 14.9 (24.1) | 15.6 (23.1) |
| *R*_free_ (%)* | 18.8 (27.3) | 16.9(23.9) | 17.2 (29.1) | 18.3 (24.6) |
| *No*. *atoms* |  |  |  |  |
| Protein | 1129 | 1141 | 1180 | 1161 |
| Ligand/ion | 46 | 41 | 42 | 42 |
| Solvent | 164 | 183 | 230 | 207 |
| B-factors (Å^2^) |  |  |  |  |
| Protein | 23.5 | 25.0 | 16.4 | 18.7 |
| Ligand/ion | 31.8 | 26.6 | 16.2 | 20.5 |
| Solvent | 35.8 | 37.3 | 31.3 | 32.0 |
| R.M.S. deviations |  |  |  |  |
| Bond length (Å) | 0.015 | 0.014 | 0.018 | 0.014 |
| Bond angle (°) | 2.24 | 2.11 | 2.18 | 2.01 |

*The numbers in parentheses are given for the highest-resolution shells.
