# Supplementary material for: Biochemical and structural characterization of an inositol pyrophosphate kinase from a giant virus
Source: EMBO J. 2024 Jan 12;43(3):7. doi: 10.1038/s44318-023-00005-0 (PMC10897400; doi:10.1038/s44318-023-00005-0)
Supplement: Supplementary file 2 — Appendix [file 44318_2023_5_MOESM2_ESM.pdf]

Appendix Figures and Table

Table of contents:

Page2	Appendix Figure S1	Alignment of putative IPKs encoded by three NCLDV genomes.
Page3	Appendix Figure S2	*Tv*IPK activity against multiple *myo*- and *scyllo*-IPs.
Page4	Appendix Table S1	DNA and Primer sequences

```
Terrestrivirus      MSITDMPNMANIANMADITNMSNIDLQSSKSVADEVIADIAEIVNKESIRIFPRIAGRSY   60
Indivirus           ------------------------------------------------MINLSQAGGHQL   12
Barrevirus          ------------------------------------------------MNNFVCIGGHQI   12
                                                                    :  :   .*:.

Terrestrivirus      IIYGQTSGIICKRMEKSDNEFVIYNYISEHYDKFLKKYVPKLYGKNND------------   108
Indivirus           I-KPLDKKI-IKPTK--YDELEFYRDYLPKYQ-ELHNCIPKFYGGGNIDEVKNMFNDDEY   67
Barrevirus          I-KIKDSRI-IKPTR--KSEVVFYKELLPKYH-NLQTFVPKYYGYGLTNDIKELFSEEEY   67
                    *     . *  *  .   .*. :*.    :*.  *:. :** ** .

Terrestrivirus      ----------MLLLEDLTYNYNNPNVMDVKIGARKRKSHTSG-------FFSIRGYTNSH   151
Indivirus           NLIINKKYEYYVILENLIDNNKIDSIIDIKLGSIHWKKNTSINEITEHKLRNVNSLTEQY   127
Barrevirus          DLILKKKYDHYIELDNLLINMTDYAILDIKLGKIHWPKNTPQKEIENHKIRNIKSTTLTH   127
                               : *::*  * .   ::*:*:*  :  .:*         : .:.. *  :

Terrestrivirus      DYKFDPDEYLTSESTINHIKNFMEAGGE--------------NRDKTKQVLLKWIMKLSE   197
Indivirus           GFRLDG--I------INNTVKYMKENCRNMKIQQIIDIIYH-LNYIHIQKIKIWINKII-   177
Barrevirus          GFRLDG--A------LVNNKIWSKEECRNMTINMIIDIFKVTLTSKSINVITDWISYLI-   178
                    .:::*          : :   : :   .                    : :  **  :

Terrestrivirus      LANDLFEINLKFDGVSLIFIYDDDCSKCDVNVVDFSRVKLIDTNDQMTISAVTNLIKILS   257
Indivirus           --NILKKINLNLYGPSLLIIISNSD--IKINLIDFAVFEESDDHNHDLIESLQNFGDVLS   233
Barrevirus          --TVLGNIDMNLYGPSILIIISGNM--VKIKLIDFTVFEETNDSLNDLIDSLTILSEIIS   234
                      . * :*:::: * *:::* ...    .::::**: .:  :   :  *.::  : .::*

Terrestrivirus      ELADNPLN   265
Indivirus           YVMLKKEN   241
Barrevirus          II------   236
                     :
```

Appendix Figure S1. Alignment of putative IPKs encoded by three NCLDV genomes.
ClustalW2 software (European Bioinformatics Institute; https://www.ebi.ac.uk/Tools/msa/clustalw2/) was used to align hypothetical proteins from *Terrestrivirus*9_15 (Accession number AYV76578.1), *Indivirus*_2_111 (Accession number ARF09732.1) and *Barrevirus*13_16 (AYV77127.1). Fully conserved residues are highlighted with an asterisk, residues with strongly similar properties are highlighted with a colon, and residues sharing weakly similar properties are highlighted with a period. Two conserved IPK functional motifs are highlighted in bold red font, D[I/V]K[I/L]G and [V/I]DF (see the main text for details).

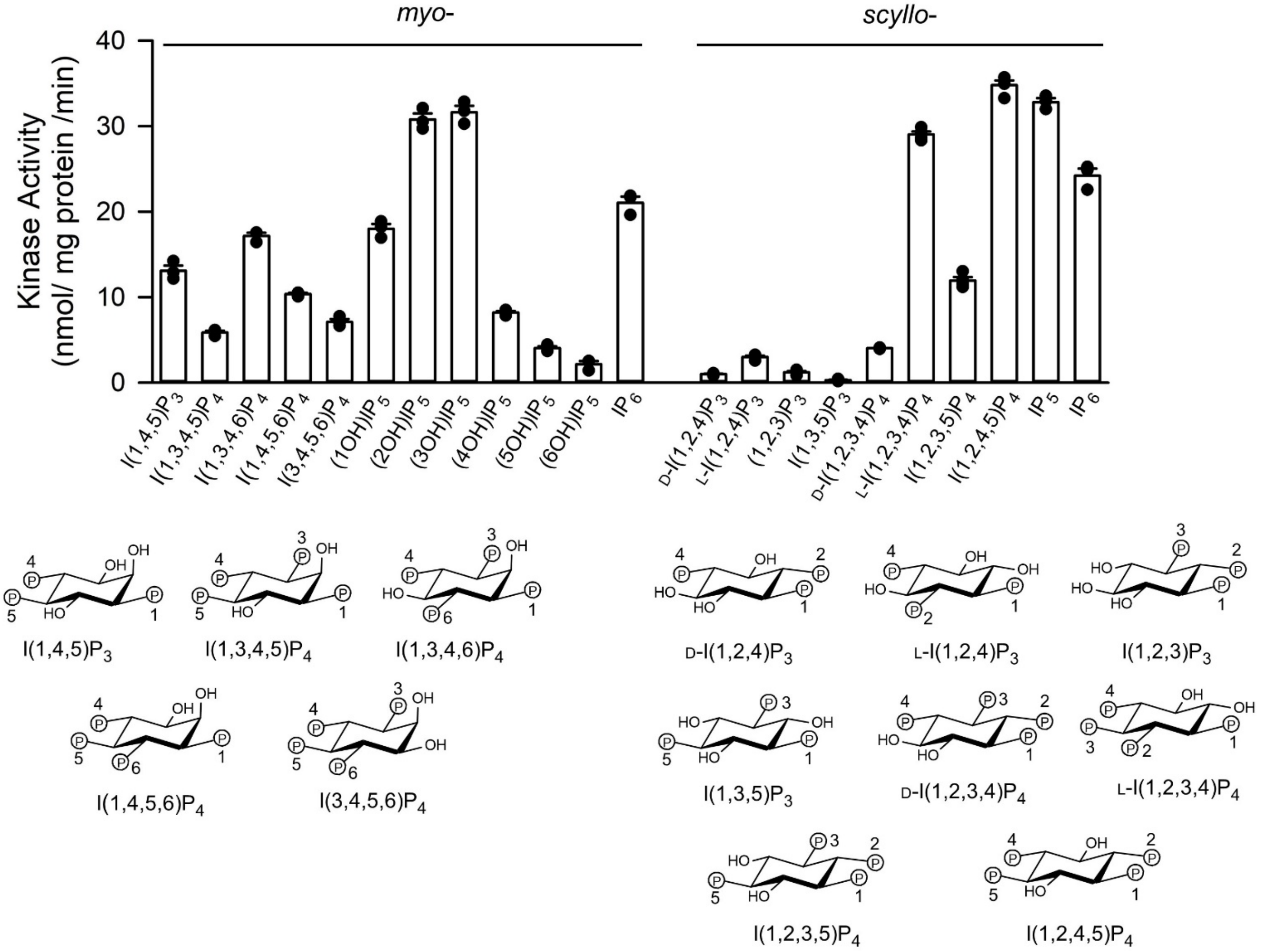

Appendix Figure S2. ***Tv*****IPK activity against multiple *myo*- and *scyllo*-IPs.**
The bar graph showing the kinase activity of *Tv*IPK towards the indicated substrates at 50 µM concentrations with 50 µM [γ-^{33}P]ATP; data represent means and standard errors (vertical bars) from either 3 or 4 independent experiments (some data points are superimposed). The structures of several substrates are shown below the bar graph; the structures of other substrates, and the nomenclature for phosphate numbering, are described in Figure 1 and its legend.

Appendix Table S1. DNA and Primer sequences

*Synthesized Tv*IPK cDNA	ATGTCAATAACAGATATGCCCAATATGGCTAACATCGCGAATATGGCCGACATCACCAACATG AGCAATATTGATCTCCAAAGCAGCAAGTCAGTTGCGGACGAAGTTATTGCGGACATCGCTGAA ATCGTGAACAAAGAAAGCATCCGCATTTTCCCGCGTATTGCCGGTCGTAGCTATATCATTTACG GCCAAACCTCTGGCATTATCTGCAAACGTATGGAAAAATCTGATAATGAGTTCGTGATCTACAA CTACATTTCCGAACATTATGACAAGTTCCTGAAGAAATACGTGCCGAAGCTGTATGGTAAAAAC AACGACATGCTGCTGTTAGAAGATCTCACGTATAACTACAACAACCCAAATGTGATGGATGTG AAAATAGGCGCACGTAAACGTAAATCGCACACCTCCGGCTTTTTCTCCATTCGCGGTTATACTA ATAGCCACGATTATAAATTCGACCCGGATGAGTACCTGACTTCCGAGAGCACCATTAATCATAT TAAGAACTTTATGGAGGCTGGTGGTGAAAACCGCGATAAGACCAAACAGGTTCTGCTGAAGTG GATTATGAAACTGAGCGAGTTGGCGAACGATTTGTTTGAGATCAACTTGAAGTTTGATGGTGTT AGCCTGATCTTCATCTACGACGACGACTGTAGCAAGTGCGATGTGAATGTTGTCGATTTTAGCA GAGTCAAGCTGATCGACACGAACGACCAGATGACCATCTCTGCAGTAACCAACTTGATTAAGA TCCTGTCGGAGTTGGCGGACAACCCGCTGAATTAA
Primers*	
Delete 1-16-F	GACATCACCAACATGAGCAATATTG
Delete 1-16-R	GGTACCGGAGCCCTGAAA
R54A-F	AAGAAAGCATCCGCATTTTCCCGGCGATTGCCGGTCGTAGCTATATCATTTACGG
R54A-R	GTAAATGATATAGCTACGACCGGCAATCGCCGGGAAAATGCGGATGCTTTCTTTG
G57A-F	GCATTTTCCCGCGTATTGCCGCGCGTAGCTATATCATTTACGGCCAAACCTCTG
G57A-R	GAGGTTTGGCCGTAAATGATATAGCTACGCGCGGCAATACGCGGGAAAATGCGG
R58A-F	TTTTCCCGCGTATTGCCGGTGCGAGCTATATCATTTACGGCCAAACCTCTGG
R58A-R	AGAGGTTTGGCCGTAAATGATATAGCTCGCACCGGCAATACGCGGGAAAATG
K76A-F	TCTGGCATTATCTGCAAACGTATGGAAGCGTCTGATAATGAGTTCGTGATCTACAACTACATTT CC
K76A-R	AAATGTAGTTGTAGATCACGAACTCATTATCAGACGCTTCCATACGTTTGCAGATAATGCCAGA GG
K128A-F	CAACAACCCAAATGTGATGGATGTGGCGATAGGCGCACGTAAACGTAAATCGC
K128A-R	GATTTACGTTTACGTGCGCCTATCGCCACATCCATCACATTTGGGTTGTTGTAG
R132A-F	AATGTGATGGATGTGAAAATAGGCGCAGCGAAACGTAAATCGCACACCTCCGG
R132A-R	GGAGGTGTGCGATTTACGTTTCGCTGCGCCTATTTTCACATCCATCACATTTG
K133A-F	GATGGATGTGAAAATAGGCGCACGTGCGCGTAAATCGCACACCTCCGGC
K133A-R	GCCGGAGGTGTGCGATTTACGCGCACGTGCGCCTATTTTCACATCCATC
R134A-F	GGATGTGAAAATAGGCGCACGTAAAGCGAAATCGCACACCTCCGGCTTTTTC
R134A-R	AAAAGCCGGAGGTGTGCGATTTCGCTTTACGTGCGCCTATTTTCACATCCATC
K135A-F	ATGTGAAAATAGGCGCACGTAAACGTGCGTCGCACACCTCCGGCTTTTTCTC
K135A-R	GAAAAAGCCGGAGGTGTGCGACGCACGTTTACGTGCGCCTATTTTCACATCC
R145A-F	CACCTCCGGCTTTTTCTCCATTGCGGGTTATACTAATAGCCACGATTATAAATTCGACCC
R145A-R	GTCGAATTTATAATCGTGGCTATTAGTATAACCCGCAATGGAGAAAAAGCCGGAGGTGTG
R234A-F	GTGCGATGTGAATGTTGTCGATTTTAGCGCGGTCAAGCTGATCGACACGAACGAC
R234A-R	CGTTCGTGTCGATCAGCTTGACCGCGCTAAAATCGACAACATTCACATCGCACTTG

* F: Forward; R: Reverse
