# Supplementary material for: Biochemical and structural characterization of an inositol pyrophosphate kinase from a giant virus
Source: EMBO J. 2024 Jan 12;43(3):7. doi: 10.1038/s44318-023-00005-0 (PMC10897400; doi:10.1038/s44318-023-00005-0)
Supplement: Supplementary file 7 — Expanded View Figures [file 44318_2023_5_MOESM7_ESM.pdf]

Expanded View Figures

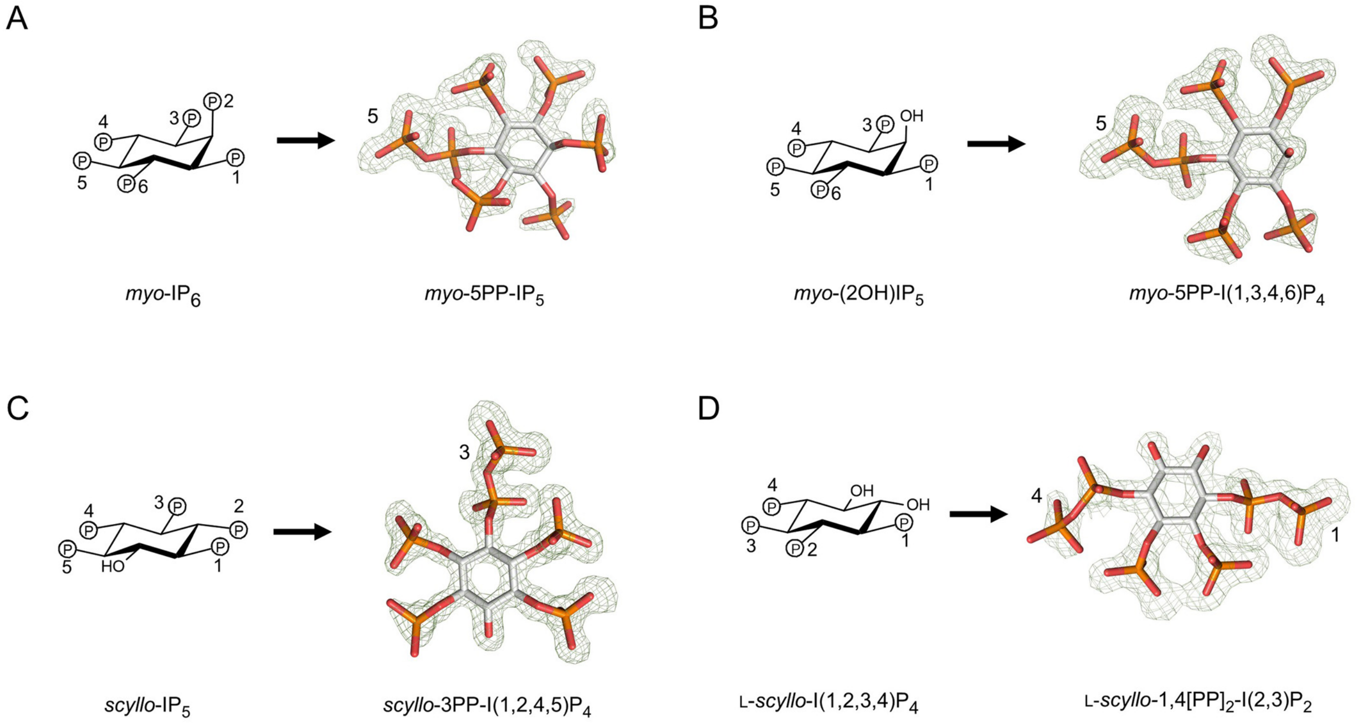

Figure EV1. Configurations of substrates captured in ADP/*Tv*IPK crystal complexes, and the structures of the corresponding products identified by their soaking into crystals of *Hs*DIPP1.

(**A–D**) substrates are shown as chemical structures, and products are shown as stick models, in which carbon is gray, phosphorous is orange, and oxygen is red. The Fo-Fc electron density maps (green mesh) are contoured at 3σ. Substituent numbering follows standard nomenclature. Each structure is aligned so that the site of *Tv*IPK phosphorylation projects to the left, with the exception of the doubly phosphorylated L-*scyllo*-1,4[PP]$_2$-(2,3)IP$_2$.

This is a U.S. Government work and not under copyright protection in the US; foreign copyright protection may apply

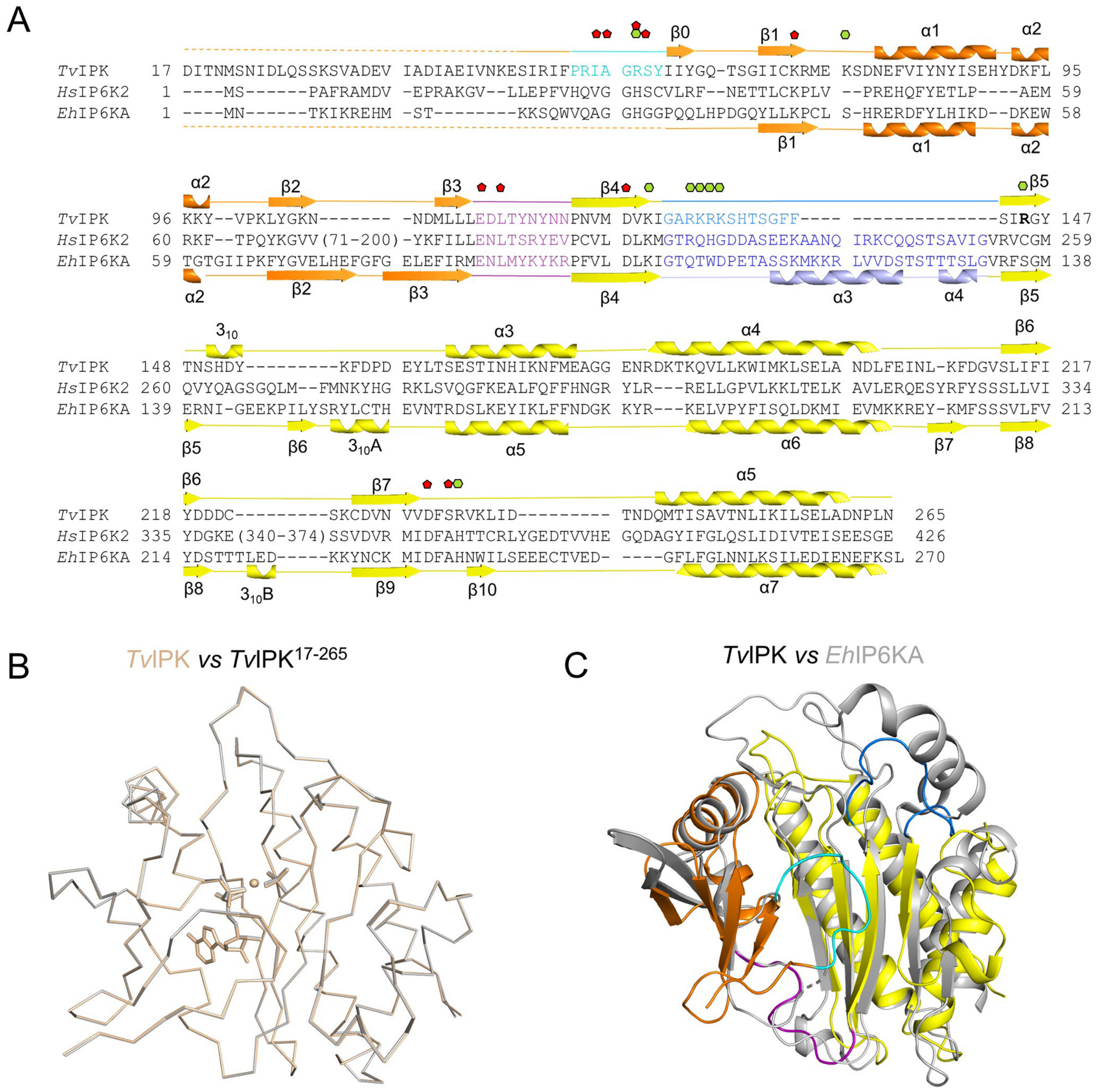

Figure EV2. Structural comparisons of *Tv*IPK with IP6Ks.

(**A**) A manually created, structure-based multiple sequence alignment of *Tv*IPK$^{17\text{-}265}$ with *Hs*IP6K2 (Accession number NP_057375.2; structural prediction by Alphafold) and *Eh*IP6KA (Accession number XP-648490.2; PBD: 4O4D). The known secondary structural elements of *Tv*IPK and *Eh*IP6KA are highlighted: α-helices and β-sheets are colored orange for their position in the N-lobe, and yellow for the C-lobe. The proposed G-loop in *Tv*IPK is colored cyan. Other known or predicted structural elements are colored as follows: hinge regions are colored purple, and the IP-binding elements are colored either light blue (for the purely loop structure in *Tv*IPK) or dark blue, for both *Hs*IP6K2 and *Eh*IP6KA. Residues that interact with substrates are highlighted with green hexagons; residues that form polar contacts with nucleotide are highlighted with red pentagons. (**B**) Superimposition of *Tv*IPK full length (colored wheat) and *Tv*IPK$^{17\text{-}265}$ (colored gray) in ribbon format; RMSD = 0.12 Å, 1589 comparable atoms). (**C**) Superimposition of *Tv*IPK (colored as Fig. 3B) and *Eh*IP6KA (colored as gray) in ribbon format. RMSD = 2.0 Å for 753 comparable atoms.

This is a U.S. Government work and not under copyright protection in the US; foreign copyright protection may apply

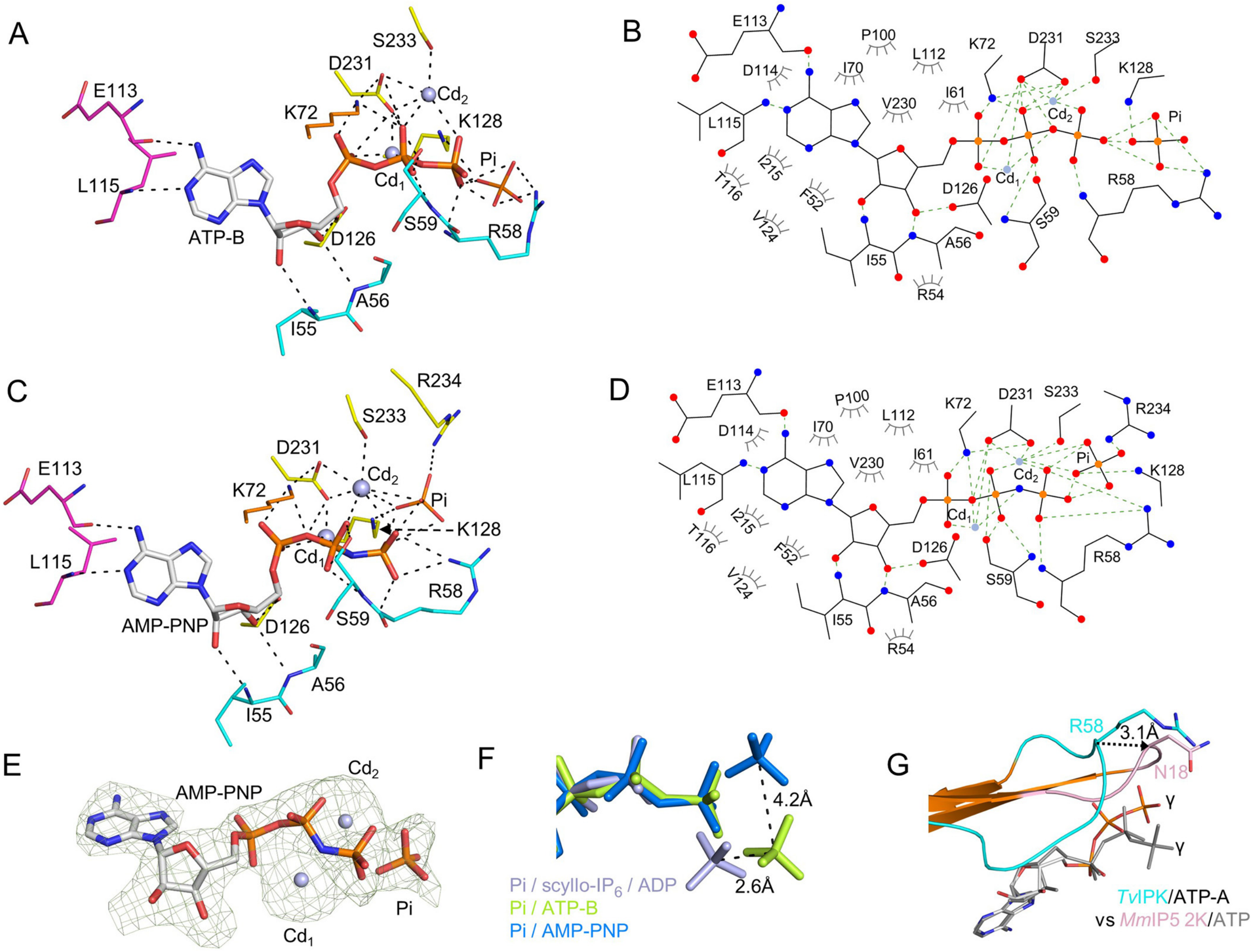

Figure EV3. The nucleotide binding site.

(**A**) Polar contacts (broken lines) of ATP in conformation B with surrounding residues, depicted in stick format. (**B**) Rendering by Ligplot+ of interactions between nucleotide and protein; Van der Waals contacts are shown as "eyelash" graphics. (**C**) Polar contacts (broken lines) of AMP-PNP with surrounding residues, depicted in stick format. (**D**) Rendering by Ligplot+ of interactions between nucleotide and protein; Van der Waals contacts are shown as "eyelash" graphics. (**E**) Omit electron density map of AMP-PNP, Cd and Pi. The Fo-Fc electron density map (green mesh) is contoured at 2.5 σ. (**F**) Superimposition of nucleotide phosphates and Pi from three *Tv*IPK structural complexes containing either Pi/scyllo-IP$_6$/ADP (light blue), Pi/ATP-B (green) or Pi/AMP-PNP (blue). (**G**) Stick model superimposition of the G-loops from *Tv*IPK (blue, in complex with ATP-A) and *Mm*IP5 2 K (green; in complex with ATP; PDB: 5MW8), represented as tubes that trace Cα atoms, except for the stick models of the indicated side chains.

This is a U.S. Government work and not under copyright protection in the US; foreign copyright protection may apply

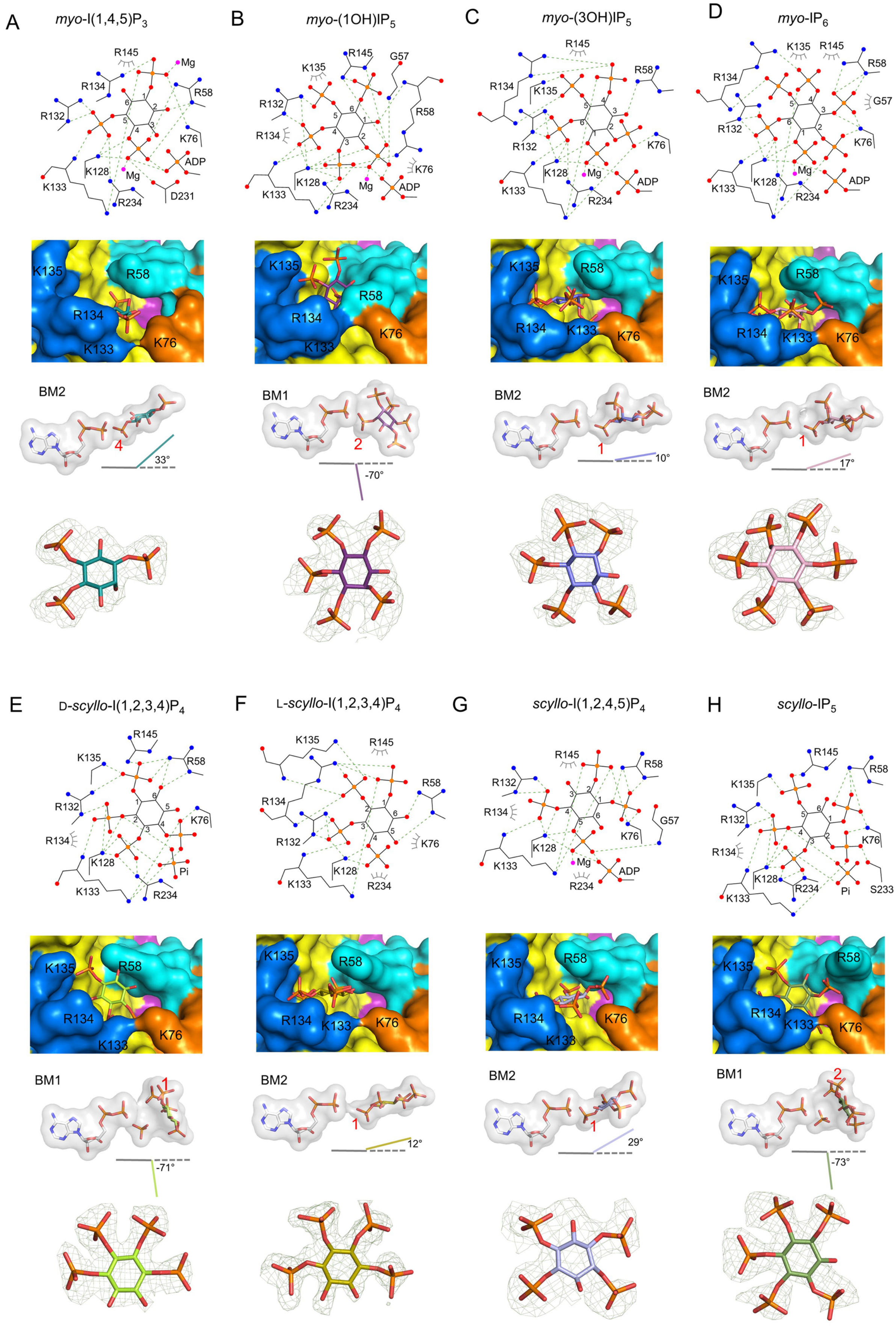
A
myo-I(1,4,5)P3
B
myo-(1OH)IP5
C
myo-(3OH)IP5
D
myo-IP6
BM2
BM1
BM2
BM2
33°
-70°
10°
17°
E
D-scyllo-I(1,2,3,4)P4
F
L-scyllo-I(1,2,3,4)P4
G
scyllo-I(1,2,4,5)P4
H
scyllo-IP5
BM1
BM2
BM2
BM1
-71°
12°
29°
-73°
R145
R134
R132
R58
K76
K128
K133
K135
R234
G57
D231
S233
Mg
ADP
Pi

Figure EV4. The characteristics of substrate binding to *Tv*IPK.

(**A–H**) Each vertical column from top to bottom depicts: a rendition created with Ligplot+ of substrate/protein interactions, *above* a space-filling depiction of the ligand binding pocket (color-coded as in Fig. 3A), above a space-filling representation of substrate and ADP (the IP phosphate closest to ADP is numbered). Note the graphical representations of the angle at which the plane of the inositol ring intersects a second plane that runs between the α- and β-phosphorus atoms of ADP through the bridging oxygen The angle of intersection between these two planes were divided into two groups (binding mode 1 and 2, i.e., BM1 and BM2). The angle for BM1 ranges from −73° to −70°); the angle for BM2 varies between +10° to +33°). The bottom panel of each column depicts the substrate's Fo-Fc electron density map (green mesh), contoured at 2.5 σ.

 This is a U.S. Government work and not under copyright protection in the US; foreign copyright protection may apply

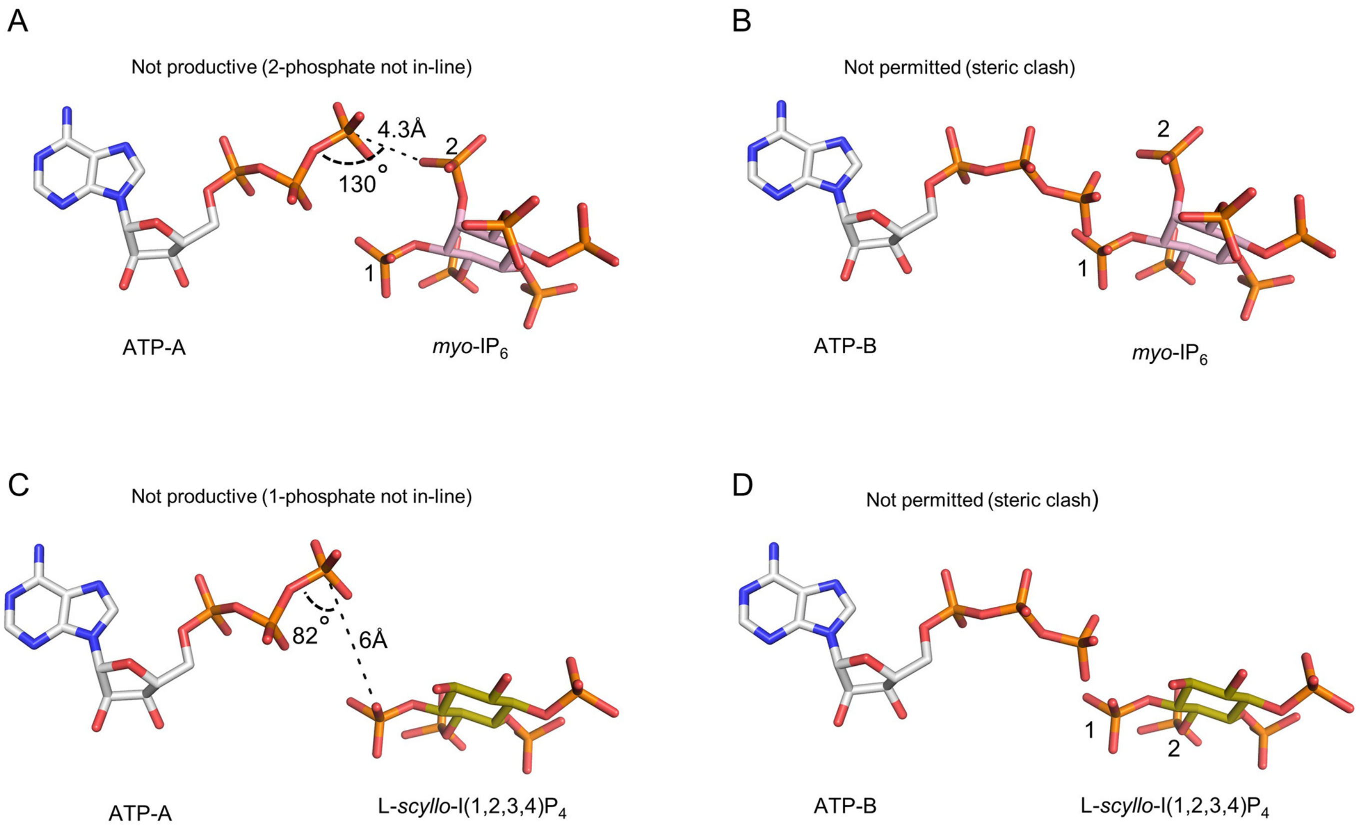

Figure EV5. Superimpositions of ATP-A and ATP-B upon the orientations of *myo*-IP$_6$ and L-*scyllo*-I(1,2,3,4)P$_4$.

(**A–D**) The configurations of ATP-A and ATP-B are taken from the *Tv*IPK/ATP complex described in Fig. 4. The configurations of *myo*-IP$_6$ and L-*scyllo*-I(1,2,3,4)P$_4$ are taken from the *Tv*IPK/ADP/substrate complexes described in Figure EV4D,F. For each of these superimpositions, the adenosine moiety has a consistent configuration and so was utilized as the reference point.

This is a U.S. Government work and not under copyright protection in the US; foreign copyright protection may apply
